# Cholinergic Elicitation Prevents Ventricular Remodeling via Alleviations of Myocardial Mitochondrial Injury Linked to Inflammation in Ischemia-Induced Chronic Heart Failure Rats

**DOI:** 10.1155/2021/4504431

**Published:** 2021-11-20

**Authors:** Yang Zhao, Huaxin Sun, Kai Li, Luxiang Shang, Xiaoyan Liang, Hang Yang, Zhenyu Dong, Jiasuoer Xiaokereti, Shuai Shang, Qina Zhou, Xianhui Zhou, Ling Zhang, Yanmei Lu, Baopeng Tang

**Affiliations:** ^1^Xinjiang Key Laboratory of Cardiac Electrophysiology and Remodeling, First Affiliated Hospital of Xinjiang Medical University, Urumqi 830011, China; ^2^Department of Pacing and Electrophysiology, First Affiliated Hospital of Xinjiang Medical University, Urumqi 830011, China; ^3^Department of Cardiology, The First Affiliated Hospital of Shandong First Medical University & Shandong Provincial Qianfoshan Hospital, Shandong Medicine and Health Key Laboratory of Cardiac Electrophysiology and Arrhythmia, Jinan, China

## Abstract

**Background:**

Cholinergic anti-inflammatory pathway (CAP) is implicated in cardioprotection in chronic heart failure (CHF) by downregulating inflammation response. Mitochondrial injuries play an important role in ventricular remodeling of the CHF process. Herein, we aim to investigate whether CAP elicitation prevents ventricular remodeling in CHF by protecting myocardial mitochondrial injuries and its underlying mechanisms.

**Methods and Results:**

CHF models were established by ligation of anterior descending artery for 5 weeks. Postoperative survival rats were assigned into 5 groups: the sham group (sham, *n* = 10), CHF group (CHF, *n* = 11), Vag group (CHF+vagotomy, *n* = 10), PNU group (CHF+PNU-282987 for 4 weeks, *n* = 11), and Vag+PNU group (CHF+vagotomy+PNU-282987 for 4 weeks, *n* = 10). The antiventricular remodeling effect of cholinergic elicitation was evaluated in vivo, and H9C2 cells were selected for the TNF-*α* gradient stimulation experiment in vitro. In vivo, CAP agitated by PNU-282987 alleviated the left ventricular dysfunction and inhibited the energy metabolism remodeling. Further, cholinergic elicitation increased myocardium ATP levels and reduced systemic inflammation. CAP induction alleviates macrophage infiltration and cardiac fibrosis, of which the effect is counteracted by vagotomy. Myocardial mitochondrial injuries were ameliorated by CAP activation, including the reserved ultrastructural integrity, declining ROS overload, reduced myocardial apoptosis, and enhanced mitochondrial fusion. In vitro, TNF-*α* intervention significantly exacerbated the mitochondrial damage in H9C2 cells.

**Conclusion:**

CAP elicitation effectively improves ischemic ventricular remodeling by suppressing systemic and cardiac inflammatory response, attenuating cardiac fibrosis and potentially alleviating the mitochondrial dysfunction linked to hyperinflammation reaction.

## 1. Introduction

Chronic heart failure (CHF), one of the most life-threatening public health problems, has resulted in many global economic and living burdens. Ischemic events such as myocardial infarction (MI) have been acknowledged as the most common etiology of CHF [1]. Due to its intricate pathogenesis, over 30% of patients with normalized pharmacological therapies or mechanotherapy still experience deteriorations in cardiac function and poor prognosis during long-term follow-up [2, 3]. Hence, it is meaningful to explore additional available therapeutic strategies.

CHF secondary to the ischemia generally results in adverse cardiac remodeling [4]. Current knowledge suggests that a sustained inflammatory response resulting from the initial myocardial injury, as well as the maladaptive cardiac energy metabolism due to mitochondrial dysfunction, jointly participate in decompensated ventricular remodeling [5–7]. Notably, chronic inflammation mediated by immune cells or proinflammatory cytokines has a close association with mitochondrial dysfunction [8]. For instance, a recent study reported that tumor necrosis factor *α* (TNF-*α*) preconditioning directly induced mitochondrial fragmentation and increased membrane potential and reactive oxygen species (ROS) overproduction in cardiomyocytes [9]. Moreover, mitochondrial-related damage accelerates inflammatory response in turn by activating the NLR family pyrin domain containing 3 (NLRP3) inflammasomes and downstream regulators of the immune signaling pathway like toll-like receptors (TLRs) [10, 11]. In short, the detrimental crosstalk between myocardial mitochondrial injury and hyperinflammation may further establish a damaging pathophysiological cycle and facilitate the ventricular remodeling [12].

The cholinergic anti-inflammatory pathway (CAP) is an endogenous self-protective mechanism connecting the autonomic neural reflex to immune regulation. Activating alpha 7 nicotinic acetylcholine receptors (*α*7nAChRs) expressed on macrophages or monocytes mediates a decrease in the transcription of downstream proinflammatory cytokines in the context of an intact vagal nerve [13–15]. Emerging evidence showed that cholinergic stimulation was able to improve cardiac remodeling and hemodynamics in the postinfarction rats, and this effect was reversed by the peripheral blockade of *α*7nAChR [16]. Other studies demonstrated the beneficial effect of activating the CAP on the arrhythmogenic substrate with ischemic cardiomyopathy, and the underlying mechanisms rely on the inflammation resolution and cardiac fibrosis attenuation [17, 18]. There are no ongoing studies regarding whether CAP elicitation ameliorates the myocardial mitochondrial injuries in the postinfarction state. Here, we aimed to study the following: (1) to verify the effect of cholinergic stimulation on ventricular remodeling especially in the cardiac energy metabolism state, in CHF models; (2) to ascertain the role of CAP activation in mitochondrial dysfunction in CHF; and (3) to uncover the potential mechanisms related to the cardioprotective effect of stimulating CAP.

## 2. Methods

### 2.1. Ethics Statement

All animal experiments were approved by the Animal Ethics Committee of the First Affiliated Hospital of Xinjiang Medical University (Approval Number: IACUC-20200318-74). All studies were conducted under the Basel Declaration and in accordance with the criteria of the Association for Assessment and Accreditation of Laboratory Care (AAALAC).

### 2.2. Animal Preparation

Eighty male purebred specific pathogen-free (SPF) Sprague-Dawley (SD) rats (5 months old), weighing 250-300 g, were purchased from the Animal Laboratory Center of Xinjiang Medical University. The animals were reared in rat cages under SPF-grade normalized laboratory conditions (12-hour light/dark cycle, 22°C, 45% humidity) with available food and water. Postoperative surviving rats were divided into 5 groups according to reduced left ventricular (LV) ejection fraction values (LVEF < 50%) with the following different interventions: sham group (thoracotomy only, *n* = 10), CHF group (coronary artery ligation for 5 weeks, *n* = 11), CHF+Vag group (CHF with right cervical vagotomy, *n* = 10), CHF+PNU group (CHF rats treated with PNU-282987, *n* = 11), CHF+Vag+PNU group (postvagotomy CHF rats treated with PNU-282987 administration, *n* = 10).

### 2.3. Surgical Procedures

#### 2.3.1. MI Establishment

All operations were carried out in a laminar flow room under aseptic conditions. Before all the invasive manipulations, each rat was generally administered 1 ml of sodium pentobarbital (2 mg/100 ml) to induce anesthesia and atropine (0.05 mg/kg) to prevent airway secretions. A multichannel physiology recorder (Lead-7000 EP CONTROL, Sichuan, China) was used to monitor the intraoperative heart rate. The anesthetized rats were fixed on a bubble brick and placed in the right lateral position. A cannula with an outer diameter of 2.5 mm (Jinkou 2.5 mm, Yu Yan Instruments, Shanghai), which was connected to the animal respirator (Ugo Basile 5025), was used for tracheal intubation with a tidal volume of 8 ml/kg. The left ventricle was exposed after thoracotomy at the left third intercostal space. The left anterior descending (LAD) branch was ligated approximately 2–3 mm below the left atrial appendage (LAA) with a 7–0 polyglactin suture. The successful MI model was determined by the ST-segment alterations from the II-lead of the ECG during a postoperative half hour. After the surgery, 50 mg of pethidine and 400 U of penicillin were administered intramuscularly for analgesia and infection prevention, respectively [19].

#### 2.3.2. Vagotomy

We subjected CHF rats to the right cervical vagotomy during anesthesia in the fifth week. The skin was incised along the right cervical line before the blunt dissection of the subcutaneous muscle layer. The right cervical vagal nerve was located in the carotid sheath, meticulously isolated by a glass needle, and cut off with ceramic scissors [20]. After the vagotomy, the incised skin was closed with interrupted sutures, and the rats were individually housed.

#### 2.3.3. Cholinergic Elicitation

PNU-282987, a selective *α*7nAChR agonist (APExBIO, No. B7007, USA) was dissolved in 0.2% DMSO organic solvent and thoroughly oscillated at 37°C until the particles had completely dissociated. Cholinergic stimulation was conducted for 4 weeks with PNU-282987 (1 mg/kg) via a slow intraperitoneal injection for 2 minutes in the CHF rats [17].

### 2.4. Echocardiography

Transthoracic echocardiography was performed weekly beginning in the first postinfarction week and lasting until the fifth week. Echocardiographic examination was implemented with a Philips HD11XE transthoracic Doppler ultrasound imaging system (Philips Inc., Bothell, WA, USA) as described in our previous work [21]. The short-axis views of the left ventricle were recorded by two-dimensional M-mode tracing at the level of the papillary muscle. The main measured indices were as follows: LV end-diastolic dimension (LVEDd), LVEF, and LV fraction shortening (LVFS). At least three consecutive cardiac beats were measured for every result.

### 2.5. Enzyme-Linked Immunosorbent Assay (ELISA)

The plasma levels of inflammatory factors, metabolic indices, and brain natriuretic peptide (BNP) were detected by ELISA. Whole blood samples were obtained from the canthus veins of rats and stored in the EDTA tubes after the completion of in vivo interventions for standby. The plasma was collected after centrifugation at 3000 r for 15 minutes. The levels of TNF-*α*, IL-1*β*, IL-6 (Multi Science Biotech, China, 70-EK382/3-96, 70-EK301B/3-96, 70-EK306/3-96), insulin (INS), BNP (Cusabio, China, CSB-E05070r, CSB-E07972r), glucose (Glu), and nonesterified fatty acid (NEFA) (Nanjing Jiancheng Bioengineering Institute, China, F006-1-1, A042-2-1) were measured with ELISA kits according to the manufacturer instructions. Absorbance (OD) value determination was performed at wavelengths of 450 nm and 570 nm within 30 minutes after the addition of termination fluid. The calibrated OD value was obtained by subtracting the measured value at 570 nm from the OD value at 450 nm.

### 2.6. Transmission Electron Microscopy (TEM)

The animals were euthanized by overdose anesthesia at the end of this experiment. Tissues were collected from the peripheral infarction area of the LV lateral wall. Each specimen was trimmed to approximately 1 mm^3^ size and directly immersed in precooled 2.5% glutaraldehyde at 4°C overnight (Sbjbio, China, SBJ-0639M). Then, the fixed specimens were subjected to gradient dehydration in an acetone solution, uranium acetate staining, and lead citrate staining as described in our previous study [22]. The ultrastructure of cardiomyocytes was observed by a JEM-100 CXII TEM (JEOL, Japan, 80 kV), and images were captured at 500x magnification.

### 2.7. Adenosine Triphosphate (ATP) Concentration Determination

Fresh tissue samples were prepared, homogenized, and centrifuged to extract the supernatant (3000 r/min, 15 minutes). The LV myocardium ATP levels were examined by an ATP assay kit (Nanjing Jiancheng Bioengineering Institute, China, A095-1-1) according to the product instructions. OD values of the tested samples were measured at a wavelength of 636 nm, and ATP concentrations were calculated based on the standard curve.

### 2.8. Histology Study

LV tissues were cut into small pieces, fixed with 4% paraformaldehyde, and embedded in paraffin. Hematoxylin-eosin (HE) staining, Masson's trichrome staining, and picrosirius red staining were performed on the 5 *μ*m thick histological sections to analyze LV fibrosis in the light of our previous method [23]. Eight paraffin blocks per group were taken for the tissue slide preparation and 9 separate bright field microscopic images per animal were quantified. The degree of fibrosis was analyzed by ImageJ software version 1.8.0. The fibrosis size was defined as the proportion of the collagen-positive area in the total myocardial area.

### 2.9. Immunohistochemistry

Tissue paraffin blocks of different groups were selected to be sliced and subjected to specimen preparation, antigen repair, and background sealing. Primary antibodies against CD68 (ab125212, Abcam, Cambridge, UK) and CD163 (ab182422, Abcam, Cambridge, UK) were added to the tissue sections and detected by the goat anti-rabbit IgG (H&L) secondary antibodies (ab150077, Abcam, Cambridge, UK). Similar to the quantitative method in histology, immunoreactivity was expressed as the entire positive staining area per square millimeter and was calculated manually by ImageJ software version 1.8.0.

### 2.10. Terminal Deoxynucleotidyl Transferase dUTP Nick End Labeling (TUNEL) Staining

LV peri-infarct myocardial tissue was embedded in paraffin and routinely sliced into 5 *μ*m sections for TUNEL staining. After a series of processes including deparaffinization, rehydration, and cell transparency, 50 *μ*l of the TUNEL mixture was added to the sections according to the instructions for the kit (No.11684817910, Roche, USA). Subsequently, the sections were incubated with converter pods for 30 minutes at 37°C, and DAB color reagent was used for chromogenic detection. Analysis of the apoptosis rate, which reflected the myocardial apoptosis level, was performed by selecting eight slides in each group randomly and then taking photos manually by laser scanning confocal microscopy (LSCM). The proportion of apoptotic cardiomyocytes was also measured using Image-Pro Plus software version 6.0.

### 2.11. Cell Culture and Treatment

H9C2(2-1) cells (Cell Bank, Chinese Academy of Sciences) were incubated in 90% DMEM medium (12800017, GIBCO) containing NaHCO_3_ (1.5 g/l) and supplemented with 10% high-quality FBS (HN-FBS-50, HAKATA). All cell lines were cultured in a 5% CO_2_ atmosphere at 37°C. Cell viability was measured by a cell growth curve, and cell proliferation was evaluated by the CCK-8 growth curve. Different concentrations of TNF-*α* (0, 5, 10, 20, 30, and 50 ng/ml) were used for choosing the optimal concentration in vitro treatment of cell lines. Untreated H9C2 cells were added as a comparison control group.

### 2.12. Flow Cytometry

#### 2.12.1. ROS Measurements

Intercellular ROS were detected by flow cytometry. Ventricular tissues were ground and filtered through a sieve to prepare a single-cell suspension. H9C2 cell lines were collected from the 6-well plates. Both the cell suspension and H9C2 cells were incubated with 2 ml phosphate-buffered saline (PBS) and 2 *μ*l of dichlorodihydrofluorescein diacetate (DCFH-DA) for 30 minutes. Next, all resuspended cells were transferred to a luciferase microplate reader (VLB000D2, Thermo Fisher) to acquire the fluorescence intensity. The optimum fluorescence excitation and emission wavelengths were set at 500 nm and 525 nm, respectively.

#### 2.12.2. Mitochondrial Membrane Potential (MMP) Measurement

After being centrifuged for 5 minutes at 1000 r, H9C2 cells were resuspended in JCI-1 staining fluid, incubated at 37°C for 20 minutes, and washed twice with precooled PBS. Then, we added 500 *μ*l cells resuspended in PBS and passed the sediments through a 200-filter mesh to prepare a single-cell suspension. TNF-*α*-induced MMP levels were assessed with 30 minutes using flow cytometry.

#### 2.12.3. Mitochondrial Permeability Transition Pore (mPTP) Measurement

The cells were lysed in a lysis buffer, preliminarily transferred, and centrifuged. Then, we added 1 ml of calcein AM staining solution to 10 ml of assay buffer, and the mixture was used to resuspend the cells. Moreover, a tube of cells in 1 ml of PBS was used as the negative control group. The single-cell suspension was prepared as previously mentioned. mPTP assessments were also achieved by flow cytometry.

#### 2.12.4. Apoptosis Evaluation

The acquisition and collection of cells from tissue and cell lines were similar to the procedures described for measuring ROS. The single-cell suspension was prepared by adding 500 *μ*l of 1x binding buffer to resuspend the cells, which were passed through a 200-filter mesh. Five microliters of Annexin V-PE and 10 *μ*l of 7-AAD were added to each tube. After gentle mixing and 10 minutes of rest at 4°C, flow cytometry was performed within 30 minutes.

### 2.13. Western Blotting

Excised ventricular tissues were promptly transferred to liquid nitrogen, and tissue homogenate was prepared. Tissue and cell samples were lysed with 200 *μ*l of RIPA lysis buffer with protease inhibitors and mixed adequately on a shaker for 1 hour. The supernatant was collected after high-speed centrifugation for 15 minutes at 15000 rpm. Protein concentrations were determined by a BCA assay kit (23235, Thermo Fisher). The quantified protein was mixed with 5x loading buffer and boiled for 5 minutes at 100°C. Electrophoresis was performed using a 10% SDS-PAGE separation gel. Semidry transfer was carried out on the PVDF membrane for 60 minutes at 100 V. Then, 5% BSA powder sealant was used to seal the membrane. After incubating overnight at 4°C with primary antibodies, we incubated the membrane with diluted secondary antibodies at room temperature for 1 hour. Finally, the chemiluminescence reagent was selected for coloration. H9C2 cells were rapidly scraped from the culture dish surface rapidly. Three tissue samples and 4 cell samples per group (25 cm^2^ cell culture flasks) were used for western blotting quantification. Every blot was conducted in duplicate. The following primary antibodies were used: anti-caspase-3 (ab184787, Abcam, Cambridge, MA, USA), anti-Bcl-2 (ab59348, Abcam, Cambridge, MA, USA), anti-Bax (ab182734, Abcam, Cambridge, MA, USA), anti-Drp1 (ab184247, Abcam, Cambridge, MA, USA), anti-Fis1 (ab71498, Abcam, Cambridge, MA, USA), anti-Mfn1 (ab221661, Abcam, Cambridge, MA, USA), anti-Mfn2 (ab124773, Abcam, Cambridge, MA, USA), anti-Opa1 (ab157457, Abcam, Cambridge, MA, USA), anti-*α*7nAChR (ab182442, Abcam, Cambridge, MA, USA), anti-NF-*κ*B (ab16502, Abcam, Cambridge, MA, USA), antiphosphorylated NF-*κ*B (ab86299, Abcam, Cambridge, MA, USA), anti-JAK2 (ab108596, Abcam, Cambridge, MA, USA), antiphosphorylated JAK2 (ab32101, Abcam, Cambridge, MA, USA), anti-STAT3 (ab68153, Abcam, Cambridge, MA, USA), and antiphosphorylated STAT3 (ab76315, Abcam, Cambridge, MA, USA).

### 2.14. Statistical Analysis

All raw data were input into Excel version 2007 and were analyzed by SPSS version 26.0. Continuous variables between the control and the TNF-*α* group are described by means ± standard deviations and were analyzed by Student's *t-*tests. Univariate ANOVA with Bonferroni post hoc comparisons was used to analyze the differences in cardiac function parameters, circulating marker levels, local immune infiltration degrees, fibrosis and apoptosis extents, and protein expression levels among the five groups. Correlations between LVEDd and the levels of IL-1*β*, IL-6, TNF-*α*, and ATP levels were analyzed by Pearson's correlation coefficient. Statistical significance was defined as a 2-tailed *P* value < 0.05.

## 3. Results

### 3.1. Cholinergic Elicitation Restored Cardiac Dysfunction and Myocardial Energy Metabolism Alterations

**Figure 1:**
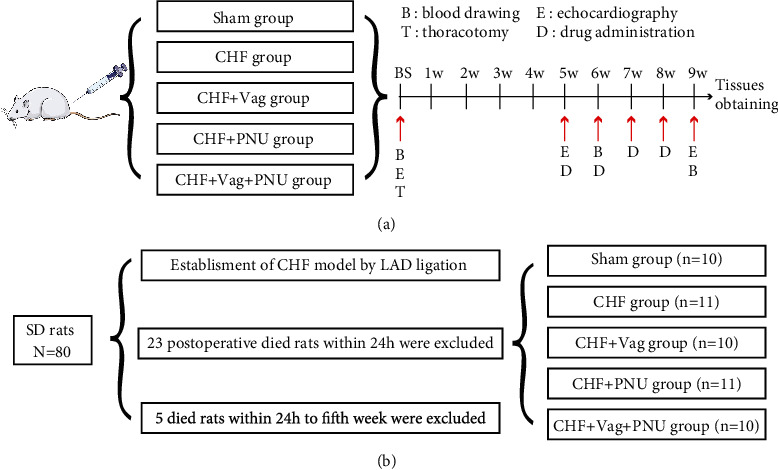
Flow chart of the experiment process: (a) study protocol diagram; (b) grouping criterion.

**Figure 2:**
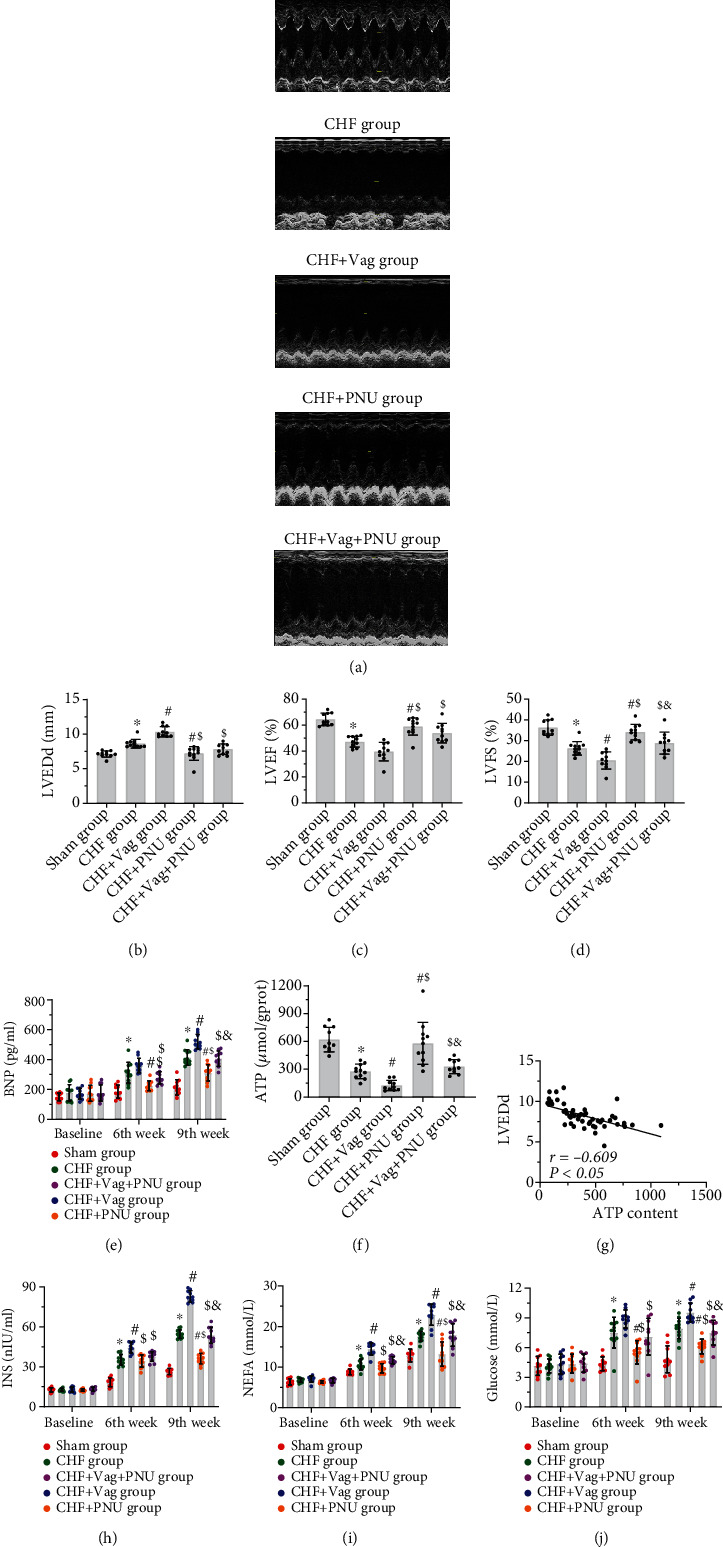
Effects of cholinergic elicitation on cardiac dysfunction and cardiac energy metabolism alterations. Representative M-mode echocardiogram of short-axis views from five groups (a). Comparisons of LVEDd (b), LVEF (c), LVFS (d), and ATP (F) among five groups. Correlation between LVEDd values of the 9^th^ week and ATP contents (g). Differences of BNP (e), INS (h), NEFA (i), and glucose (j) among five groups at baseline, the 6^th^ week, and the 9^th^ week. ^∗^*P* < 0.05 vs. Sham group. ^#^*P* < 0.05 vs. CHF group. ^$^*P* < 0.05 vs. CHF+Vag group. ^&^*P* < 0.05 vs. CHF+PNU group. LVEDd: left ventricular end-diastolic dimension; LVEF: left ventricular ejection fraction; LVFS: left ventricular fraction shortening; BNP: brain natriuretic peptide; ATP: adenosine triphosphate; INS: insulin; NEFA: nonesterified fatty acid.

All experimental procedures were conducted according to the study protocol (Figure 1(a)). In this study, the postoperative mortality of rats was approximately 30% (Figure 1(b)). Typical M-mode ultrasound schematic diagrams are shown in Figure 2(a). For echocardiographic data during the 9^th^ week, the LVEDd was enlarged in the CHF group in comparison to that in the sham group, and this parameter was further increased in the Vag group while decreased in the PNU group (*P* < 0.05, Figure 2(b)). The LVEF and LVFS values obviously declined in the Vag group. Compared to those in the CHF group, both the LVEF and LVFS values were larger in the sham and PNU groups (*P* < 0.05, Figures 2(c) and 2(d)). As shown in the Vag+PNU group, vagotomy counteracted the protective effect of PNU on LVFS but not LVEDd or LVEF (*P* < 0.05, Figure 2(d)). The exact values of the echocardiographic parameters are displayed in Supplemental Table 1. The decline in intramyocardial ATP contents in the CHF state was prevented by PNU treatment and was exacerbated by vagal mutilation (*P* < 0.05, Figure 2(f)). Correlation analysis showed that the ATP level negatively moderately correlated with LVEDd (*r* = −0.609, *P* < 0.05, Figure 2(g)). The changes in BNP, INS, NEFA, and Glu levels in the five groups showed a consistent trend with LVEDd from the 6^th^ week to the 9^th^ week, which was enhanced by vagotomy and weakened by PNU administration. Until the 9^th^ week, compared with those in the PNU group, four indicator values in the Vag+PNU group increased (Figure 2(e) and Figures 2(h)–2(j)).

### 3.2. Cholinergic Elicitation Inhibited Systemic Inflammation and Cardiac Macrophage Infiltration in the Postinfarct Phase

**Figure 3:**
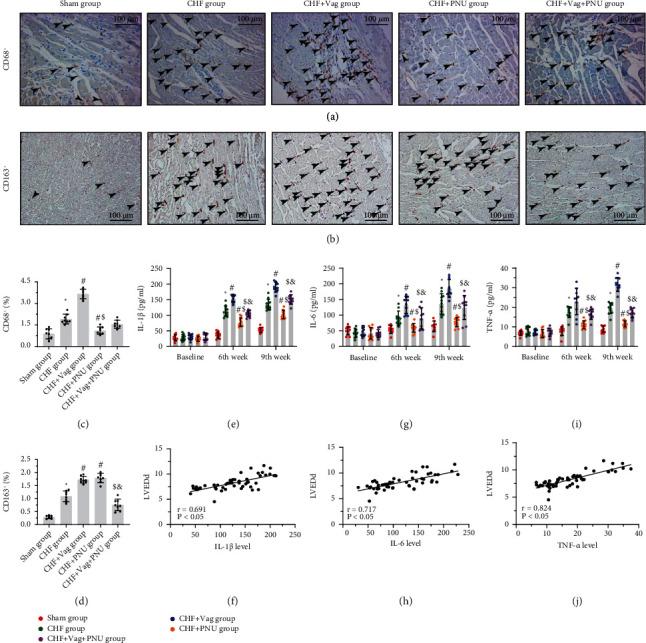
Effects of cholinergic elicitation on systemic inflammation and cardiac macrophage infiltration. Typical images of CD68^+^ staining (a) (×20) and CD163^+^ staining (b) (×20) among five groups. Quantitative analysis of immunohistochemical results (c, d). Comparisons of IL-1*β* (e), IL-6 (g), and TNF-*α* (i) among five groups at three time points at baseline, the 6^th^ week, and the 9^th^ week. Correlations of LVEDd values of the 9^th^ week with IL-1*β* (f), IL-6 (h), and TNF-*α* (j) levels of the 9^th^ week. ^∗^*P* < 0.05 vs. Sham group. ^#^*P* < 0.05 vs. CHF group. ^$^*P* < 0.05 vs. CHF+Vag group. ^&^*P* < 0.05 vs CHF+PNU group. IL-1*β*: interlukin-1*β*; IL-6: interlukin-6; TNF-*α*: tumor necrosis factor *α*.

The levels of three cytokines (IL-1*β*, IL-6, and TNF-*α*) that participate in the systemic inflammatory response are shown in Figures 3(e)–3(i). As expected, the plasma levels of these three proinflammatory factors in plasma were lowered by one week of PUN administration but were increased by vagotomy, and this trend persisted until the 9^th^ week. Pearson's correlation analysis showed the moderate positive correlations between IL-1*β*, IL-6, and TNF-*α* and LVEDd (IL-1*β*: *r* = 0.691, *P* < 0.05; IL-6: *r* = 0.717, *P* < 0.05; TNF-*α*: *r* = 0.824, *P* < 0.05, Figures 3(f)–3(j)). Immunohistochemical staining was used to evaluate the abundance of local macrophages in the myocardium. As presented in Figures 3(a) and 3(b), CD68^+^ and CD163^+^ macrophages, which represented the M1-like and M2-like phenotypes, respectively, were mostly found in the peri-infarction border areas at the 9^th^ week post-MI. Quantitative analysis demonstrated that the number of CD68^+^ macrophages increased in the Vag group and decreased in the PNU group, whereas the numbers of CD163^+^ macrophages peaked in both the Vag and PNU groups (*P* < 0.05). The impact of PNU cure on macrophage infiltration was counteracted by vagotomy to some extent (*P* < 0.05, Figures 3(c) and 3(d)).

### 3.3. Cholinergic Elicitation Suppressed Cardiac Fibrosis

**Figure 4:**
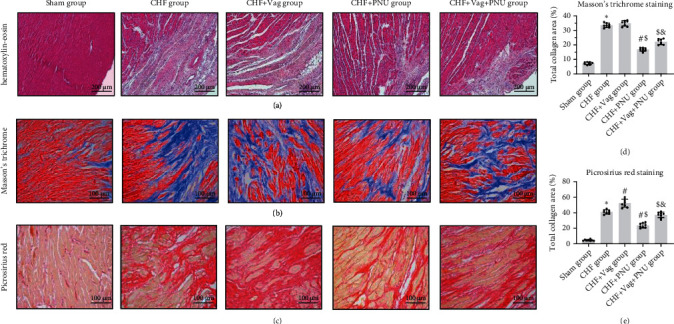
Effects of cholinergic elicitation on cardiac fibrosis. Representative images of scar ranges by HE staining in five groups (a) (×10). Masson's trichrome staining and picrosirius red staining of left ventricular noninfarct sections in five groups (b, c) (×20). Quantitative statistics of collagen contents (d, e). ^∗^*P* < 0.05 vs. Sham group. ^#^*P* < 0.05 vs. CHF group. ^$^*P* < 0.05 vs. CHF+Vag group. ^&^*P* < 0.05 vs. CHF+PNU group.

Four typical pathological changes were detected by HE staining and are shown in the Supplementary Figures online. As displayed in Figure S1A, some cardiomyocytes were covered by the accumulated inflammatory cells in a cross-sectional view. The vast formation of connective tissue in the perivascular space is presented in Figure S1B. In Figure S1C, a mass of fibrous tissue hyperplasia was accompanied by few inflammatory cells distributed around the myocardial interstitial substance. Figure S1D shows that the disrupted cardiomyocytes were replaced by the extensive production and deposition of fibrous tissue. HE staining shows scar regions in the five groups (Figure 4(a)). The Masson's trichrome staining and picrosirius red staining were performed to identify the differences in collagen deposition among the five groups. The Masson's trichrome staining showed that there were no significant differences in the total collagen areas between the CHF and Vag groups (*P* < 0.05, Figure 4(b)). However, as shown in the picrosirius red-stained sections, chronic infarction/ischemia induced collagen accumulation, which were reduced by PNU administration and were augmented by vagotomy (*P* < 0.05, Figure 4(c)).

### 3.4. Cholinergic Elicitation Alleviated Mitochondrial-Dependent Apoptosis

**Figure 5:**
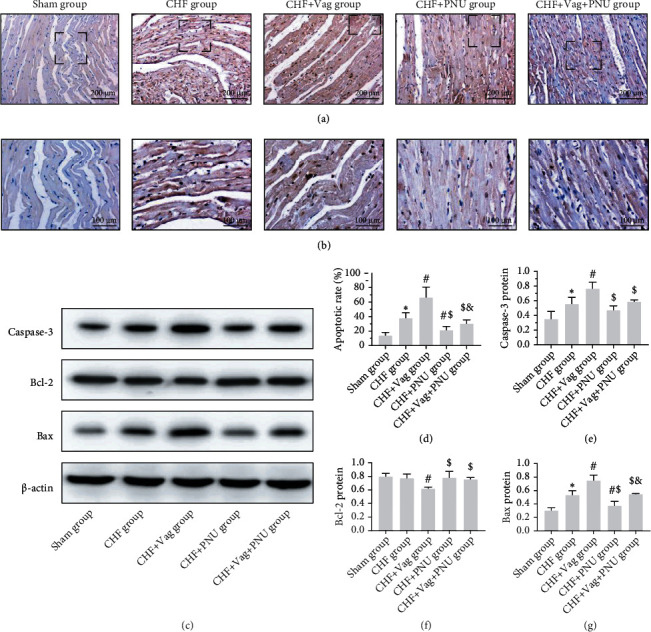
Effects of cholinergic elicitation on mitochondrial-dependent apoptosis. Representative images and detailed drawings of TUNEL staining among five groups ((a) ×10; (b) ×20). Western blot results for caspase-3, Bcl-2, and Bax of five groups (c). Quantitative statistical results of apoptotic rate (d) and relative expression levels of apoptosis-related proteins (e–g). ^∗^*P* < 0.05 vs. Sham group. ^#^*P* < 0.05 vs. CHF group. ^$^*P* < 0.05 vs. CHF+Vag group. ^&^*P* < 0.05 vs. CHF+PNU group. Bcl-2: B cell leukemia/lymphoma 2; Bax: Bcl-2-associated X.

TUNEL staining was used to assess myocardial apoptosis. As shown in Figure 5(a), the number of brown DIG-d-UTP-stained brown cells, which represented apoptotic cells, was markedly increased in CHF and Vag groups and decreased in the PNU group (*P* < 0.05, Figures 5(a) and 5(b)). Three proteins related to the mitochondrial apoptotic pathway were tested by western blotting. Quantitative analysis showed that the expression levels of caspase-3 and Bax were elevated in the CHF phase and were further upregulated by vagus transection and downregulated by PNU treatments. In contrast, the relative expression of Bcl-2 was reduced in the Vag group and augmented in the PNU group (*P* < 0.05, respectively, Figures 5(c)–5(g)).

### 3.5. Cholinergic Elicitation Attenuated the Myocardial Ultrastructural Changes

**Figure 6:**
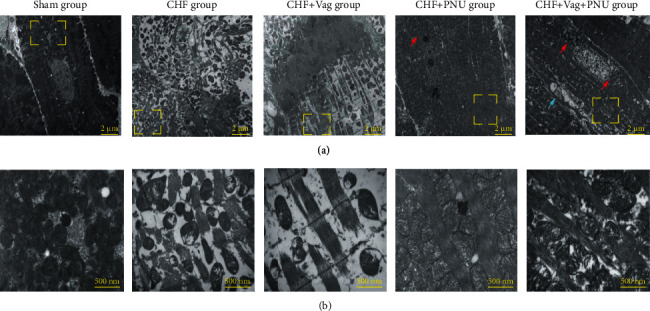
Effects of cholinergic elicitation on cardiomyocyte ultrastructural alterations. General observation (a) (×5000) and detailed view (b) (×30000) of transmission electron microscopy among five groups. Red arrow: mitophagy of autolysosome phase; green arrow: mitophagy of phagophore phase; blue arrow: mitophagy of mitophagolysosome phase.

Cardiomyocyte ultrastructural changes are shown in Figure 6. In the sham group, the well-defined mitochondria and intact sarcomeres were arranged regularly, and there were clear Z-lines. The abundant mitochondria are shown in the detailed panel. However, a panel of the CHF group showed the damaged mitochondria scattered among snatchy Z-lines tangled with the disorganized myofibers. In the CHF group, most mitochondria were present with dissociated myofibers near a few distinct lipid droplets. Similarly, a large area of impaired mitochondria accompanied by broken fibers was present in the CHF+Vag group. Furthermore, a reduction in mitochondria, the disappearance of inner and outer membranes, the absence of cristae, and mitochondrial vacuolization were clearly observed. In the CHF+PNU group, lipid droplets were surrounded by lysosomes, and most mitochondria maintained regular morphologies with slightly swollen mitochondrial cristae. Finally, the CHF+Vag+PNU group exhibited distorted cristae and discontinuous membranes.

### 3.6. Cholinergic Elicitation Blunted the Myocardial Mitochondrial Injury

**Figure 7:**
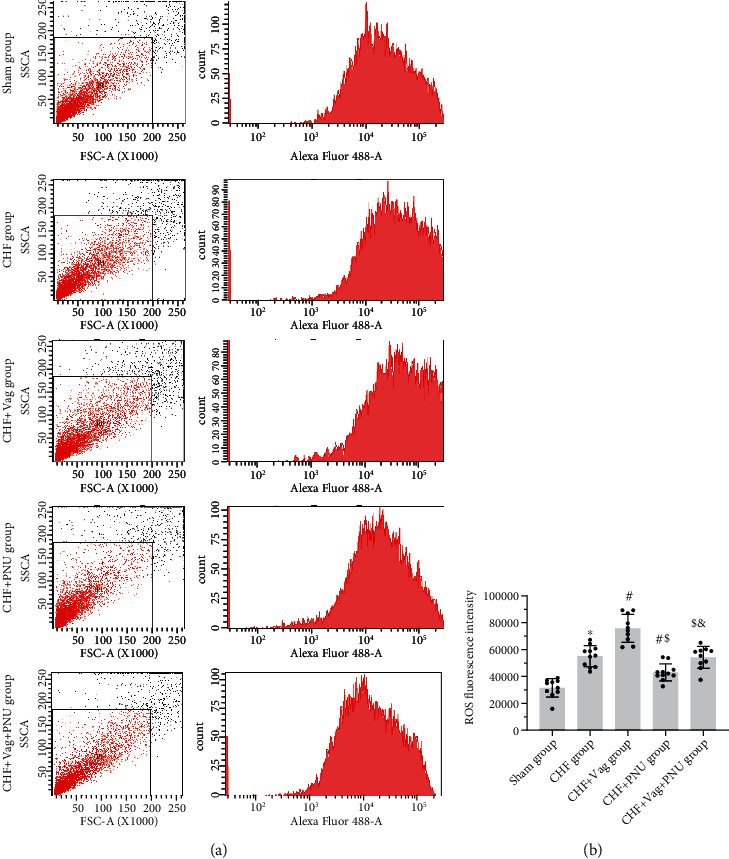
Effects of cholinergic elicitation on cardiac ROS production. Typical images of ROS production by flow cytometry among five groups (a) and the related quantitative analysis (b). ^∗^*P* < 0.05 vs. Sham group. #*P* < 0.05 vs. CHF group. $*P* < 0.05 vs. CHF+Vag group. *^&^P* < 0.05 vs. CHF+PNU group.

**Figure 8:**
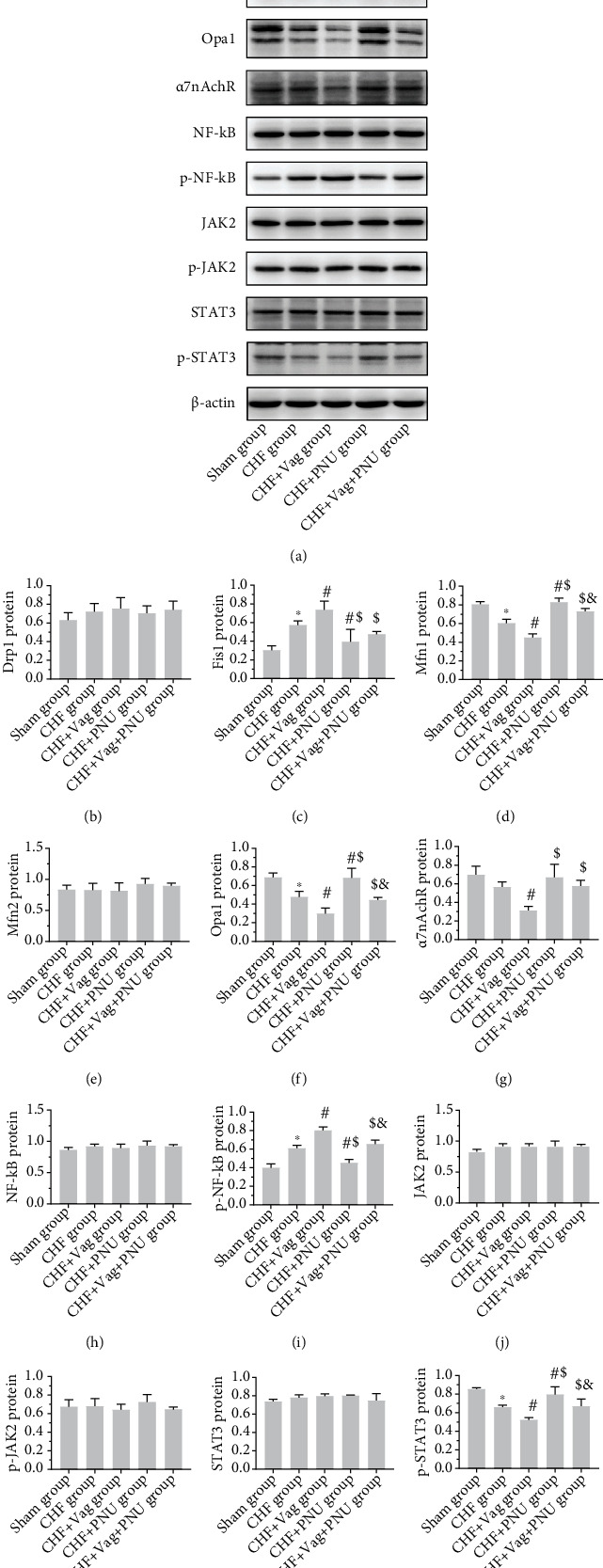
Effects of cholinergic elicitation on myocardial mitochondrial dynamics and the activities of NF-*κ*B and JAK2/STAT3 pathway. Western blot results and quantitative statistics for Drp1, Fis1, Mfn1, Mfn2, Opa1, *α*7nAChR, NF-*κ*B, P-NF-*κ*B, JAK2, P-JAK2, STAT3, and p-STAT3 (a–m). ^∗^*P* < 0.05 vs. Sham group. ^#^*P* < 0.05 vs. CHF group. ^$^*P* < 0.05 vs. CHF+Vag group. ^&^*P* < 0.05 vs. CHF+PNU group. Drp1: dynamin-related protein 1; Fis1: fission, mitochondrial 1; Mfn1: mitofusin 1; Mfn2: mitofusin 2; Opa1: OPA1 mitochondrial dynamin-like GTPase; *α*7nAChR: *α*7-nicotinic acetylcholine receptor; NF-*κ*B: nuclear factor kappa B; p-NF-KB: phosphorylated nuclear factor kappa B; JAK2: Janus kinase 2; p-JAK2: phosphorylated Janus kinase 2; STAT3: signal transducer and activator of transcription 3; p-STAT3: phosphorylated signal transducer and activator of transcription 3.

Typical flow cytometry images are shown in Figure 7(a). Quantitative analysis demonstrated that ROS levels increased in the Vag group and decreased in the PNU group (*P* < 0.05, Figure 7(b)). PNU treatment significantly downregulated mitochondrial fission-related proteins, such as Fis1, and upregulated fusion-related proteins as Mfn1 and Opa1, in comparison to those postvagotomy. However, the expression levels of other mitochondrial fission-related protein as Drp1 and fusion-related protein as Mfn2 showed insignificant differences among five groups (*P* > 0.05, Figures 8(a)–8(f)). The expression of membrane receptor *α*7nAchR was downregulated by vagotomy and was partially counteracted by PNU administration (*P* < 0.05, Figure 8(g)). Besides, vagotomy obviously lowered the expression levels of phosphorylated transcription factors NF-*κ*B and STAT3, which were rescued by PNU treatment (*P* < 0.05, Figures 8(i) and 8(m)). On the contrary, the protein levels of both NF-*κ*B, JAK2, phosphorylated JAK2, and STAT3 were not altered among five groups (*P* > 0.05, Figures 8(h) and 8(j)–8(l)).

### 3.7. TNF-*α* Mediated Mitochondrial Injury in Cardiomyocytes in a Concentration-Dependent Manner

**Figure 9:**
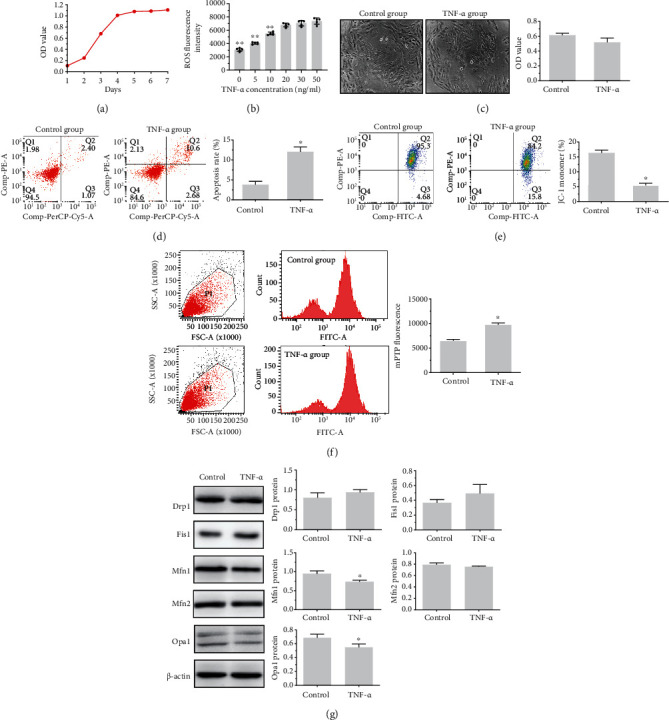
Impacts of TNF-*α* exposure on mitochondrial function of H9C2 cell lines. Evaluation of cell variability by cell growth curve (a). Screening of the optimal TNF-*α*-disposed concentration by ROS fluorescence intensity detections (b). Detections of cell proliferation by CCK-8 assay (c). Measurements of apoptosis rate, MMP, and mPTP by flow cytometry (d–f). Results of mitochondrial dynamics-related proteins between control and experimental groups by western blot (g). ^∗^*P* < 0.05 vs. control group. ROS: reactive oxygen; MMP: mitochondrial membrane potential; mPTP: mitochondrial permeability transition pore.

A TNF-*α* gradient experiment was performed to determine a target concentration for in vitro experiments. Growth curve of H9C2 cells was depicted in Figure 9(a). With increasing concentrations of TNF-*α* from 0 ng/ml to 50 ng/ml, intercellular ROS production increased (Figure 9(b)). TNF-*α* at 50 ng/ml was chosen for the optimal intervention concentration in vitro study. As anticipated, compared with the control group, TNF-*α* induced cardiomyocyte apoptosis and facilitated the reduction in MMP and opening of the mPTP without affecting cell proliferations (Figure 9(c), *P* > 0.05; Figures 9(d)–9(f), *P* < 0.05, respectively). Consistent with the western blot results at the tissue level, TNF-*α* stimulation significantly inhibited mitochondrial fusion-related markers such as Mfn1 and Opa1 (*P* < 0.05, Figure 9(g)).

## 4. Discussion

**Figure 10:**
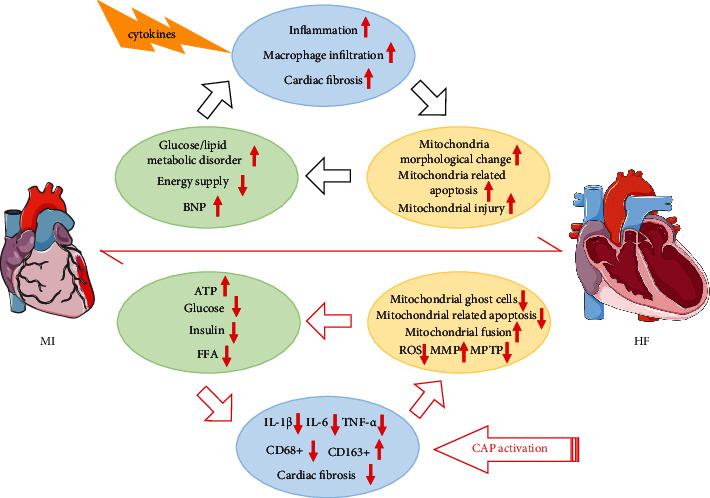
A schematic diagram of the cardioprotection of cholinergic elicitation to heart failure induced by MI. During the period of postinfarction heart advance into heart failure, chronic inflammation response induced by initial proinflammatory cytokines begets a detrimental pathophysiological circle through disturbing the mitochondrial biogenesis and eventually contributes to the ventricular remodeling. Such aforementioned vicious circle may be partly interrupted by cholinergic elicitation.

The major findings of the present study were as follows: (1) CAP activation ameliorated the impaired cardiac function, reduced the LV dilatation, and restored abnormal Glu and lipid metabolism in the chronic infarction phase; (2) CAP induction mitigated systemic and local cardiac inflammatory response and suppressed myocardial fibrosis; (3) CAP elicitation prevented the mitochondrial dysfunction by inhibiting mitochondria-dependent apoptosis, ameliorating ROS overload, and promoting mitochondrial fusion; and (4) the phosphorylation of STAT3 and NF-*κ*B in cardiomyocytes was involved in the cardioprotective effect of positively modulating the CAP (Figure 10).

Clinically, CHF challenged by MI tends to be a consequence of cardiac pathologic remodeling during the postinfarction late phase [24]. First, in order to simulate the pathophysiological features of ischemic HF, we established the postinfarction CHF models, which successfully mimicked the hallmarks including the altered ventricular structure, the impaired pump function, and disordered energy metabolism after a five-week cardiac remodeling period [25, 26].

Then, in order to examine the macroscopic effect of CAP modulation on cardiac dysfunction and energy metabolism state, we bidirectionally manipulated CAP activities with PNU treatment and vagotomy in the 6^th^ week. Coinciding with previous results regarding the salutary effect of cholinergic induction on cardiac dysfunction [27–29], we demonstrated that activating the CAP improved ventricular structural remodeling and mechanical function, as evidenced by echocardiographic parameters and BNP levels. For the first time, to our knowledge, we reported that the levels of INS, NEFAs, and Glu continuously increased in CHF rats, which was partly inhibited by CAP stimulation and exacerbated by vagotomy. To explain this result, physiological evidence suggested that enhanced lipolysis induced by hypersympathetic activities worsened the reduction in Glu uptake and increased insulin resistance in advanced HF [30]. It was verified that cholinergic elicitation can modulate autonomic nerve activity and especially suppress sympathetic activities in many cases [31, 32]. In addition, we found that insufficient ATP levels were moderately correlated with LV expansion, and an improved energy supply was achieved by targeting the CAP. From these observations, we hypothesized that mitochondria, which link substrate utilization and ATP production, may be essential for the protective effect of cholinergic stimulation.

Next, we focused on the three key pathophysiological responses to HF progress (inflammation, fibrosis, and mitochondrial injury) to describe the detailed beneficial effects of cholinergic elicitation. Solid evidence suggests that a prolonged inflammatory response challenged by myocardial injury, namely, chronic inflammation, is implicated in pathological cardiac remodeling and also has the crosstalk with cardiac fibrosis and mitochondrial injuries [33–35]. In our study, the systemic inflammatory reaction in the postinfarction stage was mitigated by activating the CAP but intensified by unilateral vagotomy, as proven by serum proinflammatory cytokine levels. The levels of IL-1*β*, IL-6, and TNF-*α* changed from the first week after intervention, which suggested a rapid anti-inflammatory effect of cholinergic stimulation. However, circulatory cytokine levels are susceptible to the anti-inflammatory effect of peripheral organs when the CAP is activated and fails to reflect the regional cardiac inflammation [36, 37]. Thus, immunohistochemistry was performed to examine cardiac macrophage infiltration. In a prior study, stimulating the CAP was shown to promote the transformation of Ly-6C^high^ macrophages to Ly-6C^low^ macrophages in the late infarction phase [17]. Similarly, our results showed that cholinergic evocation reduced the accumulation of M1 macrophages and increased M2 macrophages infiltration in the peri-infarct area. A reasonable explanation for this result is that anti-inflammatory cytokines released from M2 macrophages limit the activation and proliferation of M1 macrophages [38]. Interestingly, a question originating from our study is why M1 and M2 macrophages maintain the high abundances in the Vag group. This effect may occur because the biological functions of recruited macrophages at injured sites as proinflammation or repair are dependent on microenvironmental stress [39]; that is, the enhanced macrophage plasticity synchronously tends to the proinflammation and anti-inflammation preponderances in the absence of vagus [40]. In terms of the pathological results, upregulating the CAP ameliorated cardiac fibrosis in the peri-infarction area, while vagus transection partially offsets this protective effect. Previous evidence supported that macrophage polarization plays an important role in the process of cardiac fibrosis [41]. In our study, the decrease in M1 macrophages induced by cholinergic elicitation may mediate attenuation of extracellular matrix remodeling by downregulating the expression of matrix metalloproteinase, TNF-*α*, and IL-1 [42].

Most importantly, we reported the ameliorative effect of CAP activation on mitochondrial injuries under CHF conditions for the first time. In the context of chronic inflammation, detrimental cytokines such as TNF-*α* trigger mitochondrial dysfunction via inducing ROS overproduction and further beget a series of downstream events as apoptosis and hyperinflammation [8]. Mitochondria-mediated apoptosis relies on the inhibition of Bcl-2 and the activation of Bax and caspase-3/7 [43]. Here, we observed a decline in ROS overload in the PNU group and the alleviation of cardiomyocyte apoptosis, especially the mitochondria-mediated apoptosis, according to changes in mitochondrial apoptosis-related proteins such as caspase-3, Bcl-2, and Bax. Different periods of ongoing mitophagy were discovered by TEM, including the segregation of damaged mitochondria and degradation mediated by lysosomes. Regrettably, no further investigation was conducted in allusion to mitophagy in the present study. It has been widely reported that steady mitochondrial dynamics confer extensive advantages to mitochondrial biogenesis and cellular events [44]. Thereupon, we detected the changes in key proteins implicated in mitochondrial dynamics under CAP initiation. Confusingly, the expression of both Mfn1 and Opa1 was upregulated in the PNU group and downregulated in the Vag group, while Fis1 but not Drp1 expression changed after cholinergic interventions. We merely speculated that CAP induction potentially drove more healthy mitochondrial fragments toward rebirth through mitochondrial fusion mediated by Mfn1 and Opa1 [45], but the exact mechanisms remain unclear.

In consideration of the beneficial effect of CAP elicitation, numerous studies have focused on the activated JAK/STAT3 pathway and NF-*κ*B/HMGB1/RAGE pathway in immune cells [46]. Our study suggested that the activation of STAT3 and inactivation of NF-*κ*B were implicated in the cardioprotective effects of CAP stimulation, possibly due to the negative modulations of these two transcription factors on downstream phenotypes like inflammation, apoptosis, and MPTP activities, through the signaling cascade triggered by TNF-*α*/TNFR [47].

Finally, an in vitro cell experiment was performed to validate the association of mitochondrial injury with proinflammatory cytokines. As expected, together with the reductions in MMP and mPTP opening, the major event of mitochondrial apoptosis [48] was increased markedly when cells were exposed to the exogenous TNF-*α*. These results are consistent with other findings, as evidenced by the enhanced mitochondrial apoptosis in the CHF and Vag groups in vivo. Consistently, the expression of mitochondrial fusion-related proteins as Mfn1 and Opa1 was downregulated when challenged by TNF-*α*. However, we observed that TNF-*α* had no effect on cell proliferations. The present in vitro experiment may imitate a systemic scenario in which hyperinflammation contributes to mitochondrial dysfunction and accelerates a vicious pathophysiological cycle.

## 5. Limitations

There were a few limitations in our study. First, to highlight the protective effect of cholinergic elicitation at any one time point, we ignored the influences of time factor on several repeated measurement data and used univariate ANOVA to analyze effects of indicators such as metabolisms and cytokines. Second, the method of CAP modulation was based on the methods of previously published work, and it was still unclear whether the different degrees of activating or inhibiting the CAP had different effects on the expected outcome. Third, the results of the cell experiment only explained the detrimental impact of exogenous proinflammatory cytokine on mitochondria and indirectly indicated that eliciting the CAP prevented mitochondrial injury linked to inflammation. However, a better design should be aimed at investigating the protective effect of CAP induction on HF cell models. Fourth, our results merely affirmed the protective role of CAP stimulation on CHF induced by MI, but the therapeutic effects on cardiac remodeling caused by other pathogenic factors, such as hypertrophy and cardiotoxicity, remain unknown. Fifth, our results were inclined to the onefold observations of efficacy produced by eliciting CAP and the descriptions of altered pathophysiological phenomena, whereas the key target of cholinergic stimulation in ameliorating mitochondrial dysfunction and the exact molecular mechanisms were still not elucidated.

## 6. Clinical Implications

Two emerging clinical trials announced that the applications of IL-1*β* inhibitors and sodium glucose cotransporter-2 inhibitors significantly lowered the hospitalization for HF and all-cause mortality among CHF patients. These inspiring results indicated the feasibility and potential use of anti-inflammatory agents and improved cardiac energy metabolism therapies in CHF fields [49–51]. Certainly, the anti-inflammatory effects of CAP elicitation against various diseases, such as rheumatoid arthritis and ulcerative colitis, have been proven by strong evidence, but few researchers have focused on cholinergic activation to cure CHF [52]. Numerous treatment strategies targeting the CAP including neuromodulation as well as drug administration have been preliminarily applied to clinical practice and have shown the helpful curative effects in cardiovascular diseases [53–55]. Spurred by our promising results, further studies should focus more on the clinical translation of cholinergic elicitation in CHF populations.

## 7. Conclusion

This study provides evidence that cholinergic elicitation exerts a salutary effect by suppressing the production of proinflammatory cytokines, attenuating cardiac fibrosis, and potentially ameliorating inflammation-linked myocardial mitochondrial injuries. These alterations above are associated with the holistic inflammation resolution, systemic energy metabolism balance, and improvements in cardiac dysfunction.

## Data Availability

The datasets of this study are available from the corresponding authors on demand.

## Supplementary Materials

Supplementary 1 Figure S1: typical pathologic changes in the myocardium. Inflammation infiltration (A); perivascular fibrosis (B); reactive fibrosis (C); reparative fibrosis (D).

Supplementary 2 Table 1: ∗*P* < 0.05 vs. sham group at the same point in time, ^#^*P* < 0.05 vs. CHF group at the same point in time. LVEDd: left ventricular end-diastolic diameter; LVEDs: left ventricular end-systolic diameter; LVPWd: left ventricular posterior wall thickness at the end of diastole; LVPWs: left ventricular posterior wall thickness at the end of systole; LVEF: left ventricular ejection fraction; LVFS: left ventricular fraction shortening.
