# Supplementary material for: Cholinergic Elicitation Prevents Ventricular Remodeling via Alleviations of Myocardial Mitochondrial Injury Linked to Inflammation in Ischemia-Induced Chronic Heart Failure Rats
Source: Mediators Inflamm. 2021 Nov 20;2021:4504431. doi: 10.1155/2021/4504431 (PMC8627564; doi:10.1155/2021/4504431)
Supplement: Supplementary 2 — Table 1: ∗P < 0.05 vs. sham group at the same point in time, #P < 0.05 vs. CHF group at the same point in time. LVEDd: left ventricular end-diastolic diameter; LVEDs: left ventricular end-systolic diameter; LVPWd: left ventricular posterior wall thickness at the end of diastole; LVPWs: left ventricular posterior wall thickness at the end of systole; LVEF: left ventricular ejection fraction; LVFS: left ventricular fraction shortening. [file 4504431.f2.pdf]

Supplemental table 1

Groups	LVEDd,mm	LVEDs,mm	LVPWd,mm	LVPWs,mm	LVEF, %	LVFS, %
**Sham**						
baseline	6.65±0.33	4.61±0.6	1.65±0.22	2.23±0.32	60.04±13.78	30.63±8.55
5 weeks	6.98±0.42	4.59±0.52	1.67±0.23	2.24±0.32	61.56±7.15	34.43±4.78
9 weeks	7.13±0.45	4.63±0.52	1.79±0.28	2.24±0.34	63.38.90±4.82	36.31±3.76
**CHF**						
baseline	6.46±0.27	4.38±0.39	1.70±0.25	2.19±0.24	58.97±7.32	32.2±5.05
5 weeks	7.75±0.93*	6.01±0.75*	1.87±0.23*	2.28±0.22	43.62±5.49*	22.47±3.34*
9 weeks	8.63±0.63*	6.18±0.69*	2.15±0.2*	2.59±0.31*	47.19±4.09*	26.33±3.28*
**CHF+VAG**						
baseline	6.46±0.34	4.72±0.54	1.64±0.34	2.13±0.22	59.87±15.95	26.64±9.58
5 weeks	8.29±0.74*	6.47±0.91*	1.83±0.33	2.32±0.32	42.83±8.52*	22.19±5.12*
9 weeks	10.35±0.75*#	8.00±0.95*#	2.28±0.34*	2.61±0.32*	39.57±7.17*	20.51±4.17*#
**CHF+PNU**						
baseline	6.61±0.43	4.48±0.51	1.65±0.28	2.17±0.26	58.39±11.83	32.16±7.94
5 weeks	8.17±0.29*	6.27±0.65*	2.00±0.23*	2.51±0.17*	44.25±12.65*	23.27±7.68*
9 weeks	7.20±1.01#	5.21±0.63*#	1.71±0.19#	2.20±0.38#	58.91±6.68#	34.2±3.73*
**CHF+Vag+PNU**						
baseline	6.29±0.36*	4.60±0.72	1.75±0.15	2.10±0.23	58.41±15.83	26.88±9.83
5 weeks	7.36±0.56	5.98±0.64*	1.88±0.16	2.41±0.33	37.31±10.56*	18.84±6.0*
9 weeks	7.83±0.76*#	5.41±0.44*#	1.82±0.13#	2.3±0.31	53.82±7.52*	28.89±5.28*

*$P<0.05$ vs sham group at the same point in time, #$P<0.05$ vs CHF group at the same point in time. LVEDd, left ventricular end-diastolic diameter; LVEDs, left ventricular end-systolic diameter; LVPWd, left ventricular posterior wall thickness at the end of diastole; LVPWs, left ventricular posterior wall thickness at the end of systole; LVEF, left ventricular ejection fraction; LVFS, left ventricular fraction shortening.
